# Phylogenomics and Genetic Diversity of *Arnebiae Radix* and Its Allies (*Arnebia*, Boraginaceae) in China

**DOI:** 10.3389/fpls.2022.920826

**Published:** 2022-06-09

**Authors:** Jiahui Sun, Sheng Wang, Yiheng Wang, Ruishan Wang, Kangjia Liu, Enze Li, Ping Qiao, Linyuan Shi, Wenpan Dong, Luqi Huang, Lanping Guo

**Affiliations:** ^1^State Key Laboratory Breeding Base of Dao-di Herbs, National Resource Center for Chinese Materia Medica, China Academy of Chinese Medical Sciences, Beijing, China; ^2^Laboratory of Systematic Evolution and Biogeography of Woody Plants, School of Ecology and Nature Conservation, Beijing Forestry University, Beijing, China

**Keywords:** *Arnebiae Radix*, *Arnebia euchroma*, *Arnebia guttata*, chloroplast genome, genetic diversity, phylogeography, phylogenomics

## Abstract

*Arnebiae Radix* is a traditional medicine with pleiotropic properties that has been used for several 100 years. There are five species of *Arnebia* in China, and the two species *Arnebia euchroma* and *Arnebia guttata* are the source plants of *Arnebiae Radix* according to the Chinese Pharmacopoeia. Molecular markers that permit species identification and facilitate studies of the genetic diversity and divergence of the wild populations of these two source plants have not yet been developed. Here, we sequenced the chloroplast genomes of 56 samples of five *Arnebia* species using genome skimming methods. The *Arnebia* chloroplast genomes exhibited quadripartite structures with lengths from 149,539 and 152,040 bp. Three variable markers (*rps16-trnQ*, *ndhF-rpl32*, and *ycf1b*) were identified, and these markers exhibited more variable sites than universal chloroplast markers. The phylogenetic relationships among the five *Arnebia* species were completely resolved using the whole chloroplast genome sequences. *Arnebia* arose during the Oligocene and diversified in the middle Miocene; this coincided with two geological events during the late Oligocene and early Miocene: warming and the progressive uplift of Tianshan and the Himalayas. Our analyses revealed that *A. euchroma* and *A. guttata* have high levels of genetic diversity and comprise two and three subclades, respectively. The two clades of *A. euchroma* exhibited significant genetic differences and diverged at 10.18 Ma in the middle Miocene. Three clades of *A. guttata* diverged in the Pleistocene. The results provided new insight into evolutionary history of *Arnebia* species and promoted the conservation and exploitation of *A. euchroma* and *A. guttata.*

## Introduction

*Arnebiae Radix* is a traditional Persian, Unani, Ayurvedic, and Chinese medicine with pleiotropic properties that has been used for several 100 years (Zhu, 1982; Kumar et al., 2021). *Arnebiae Radix* is an ingredient in 122 Chinese patent medicines and 195 Chinese medicine prescriptions. Shikonin, shikonofurans, and red naphthoquinones are the main effective chemical constituents, and they have been widely used for the treatment of infections, inflammation, and bleeding for their anti-inflammatory, anti-fungal, and anti-angiogenic activities (Gupta et al., 2014; Feng et al., 2020; Kumar et al., 2021). Shikonin and its derivatives exhibit anti-cancer and anti-tumorigenic activities and have the potential to be used for the development of anti-tumor drugs (Feng et al., 2020; Liao et al., 2020; Kumar et al., 2021). *Arnebiae Radix* has also been used in the printing, dyeing, and cosmetics industries (Ma et al., 2021). Although *Arnebiae Radix* is widely used for its varied medicinal effects, the evolutionary history of its source plants remains unclear.

*Arnebiae Radix* is the dried root of *Arnebia euchroma* (Royle ex Benth.) I. M. Johnst. and *Arnebia guttata* Bunge according to the Chinese Pharmacopoeia (2020 version). *Arnebia* Forsskål is a genus in the family Boraginaceae and the tribe Lithospermeae. *Arnebia* comprises ca. 25 species and are distributed from North Africa to Central Asia and the Himalayas (Johnston, 1954). Only six *Arnebia* species to date have been reported from China (Zhu, 1982), including *A. euchroma*, *A. guttata*, *A. decumbens* (Ventenat) Cosson & Kralik, *A. tschimganica* (B. Fedtschenko) G. L. Chu, *A. szechenyi* Kanitz, and *A. fimbriata* Maximowicz. *A. guttata* (Inner Mongolia *Arnebiae Radix*) mainly occurs in Xizang, Xinjiang, western Gansu, Ningxia, and Inner Mongolia in China. *A. euchroma*, commonly known as Xinjiang *Arnebiae Radix*, is an endangered herb (Kala, 2000; Gupta et al., 2014) that grows naturally on the slopes and dry patches in cold desert temperate zones of the western Himalayas (Xinjiang, western Xizang) (Lal et al., 2020). *A. szechenyi* inhabits sunny rocky slopes and sand dune edges; most populations of this species occur in areas surrounding the Tengger Desert in Northwest China (Fu et al., 2021). *A. decumbens* is an annual plant, and its native range extends from North Africa to Mongolia, including the Arabian Peninsula; it is also native to the Canary Islands. In China, its range is restricted to northern Xinjiang. *A. fimbriata* has a densely gray–white hirsute and is distributed in western Gansu, Ningxia, Qinghai, and Mongolia. *A. tschimganica* is an endangered herb in China (Qin et al., 2017). Recent studies have shown that this species belongs to the monotypic *Ulugbekia* [*Ulugbekia tschimganica* (B. Fedtsch.) Zakirov] (Weigend et al., 2009).

Owing to its various medicinal properties, *Arnebia* species have been overexploited, and the populations of some species have declined so much. There are two major outstanding problems regarding the exploitation and conservation of *Arnebiae Radix* and its source plants that need to be addressed. First, the sources of the medicinal materials of *Arnebiae Radix* in the market are complex according to one field study (Liu et al., 2020). Identification of source species is difficult based on morphological features; there is thus an urgent need to develop more efficient methods for species identification, such as DNA barcoding (Liu et al., 2020; Xu et al., 2021). Second, a sound understanding of patterns of genetic diversity and genetic divergence among wild plant populations is important for plant seeding and the conservation of threatened species (Funk et al., 2012). However, few studies have evaluated the population genetics of the source plants of *Arnebiae Radix*. Improved genetic markers need to be developed to facilitate the identification of the source species of *Arnebiae Radix* and clarify the population genetics of *A. euchroma* and *A. guttata*.

Chloroplasts are key plant organelles that are involved in photosynthesis and important biological processes, such as fatty acid and amino acid synthesis (Sugiura, 1992). The chloroplast genome of higher plants is a double-stranded and circular DNA molecule with a typical quadripartite structure (Dong et al., 2013, 2021a; Villanueva-Corrales et al., 2021), including a pair of inverted repeats (IRs) as well as a large single copy (LSC) and a small single copy (SSC) region. The chloroplast genome encodes approximately 80 protein-coding, 30 transfer RNA (tRNA), and four ribosomal RNA (rRNA) genes, and the structure of the chloroplast genome, including the gene content and gene order, is conserved. An increasing number of chloroplast genomes are being sequenced due to the development of sequencing technology and genome assembly methods.

The nucleotide substitution rate of the chloroplast genome is moderate compared with that of the nuclear genome; it is also uniparentally inherited and exhibits low rates of recombination (Schwarz et al., 2017; Dong et al., 2020). Chloroplast genome sequences are thus effective for inferring phylogenetic relationships at various levels of divergence (Dong et al., 2018, 2022b), characterizing population structure (Qiao et al., 2020; Xiao et al., 2021), identifying species (Ślipiko et al., 2020; Dong et al., 2021b), and elucidating patterns of genome evolution and molecular evolution (Dong et al., 2020). The chloroplast genomes of *Arnebia* have been compared with those of *Lithospermum* (Park et al., 2020); the full chloroplast genome sequences of *A. guttata* and *A. euchroma* have been published, and molecular markers have been developed at the genus level through comparative analysis. However, interspecific and intraspecific variation among *Arnebia* species has not yet been clarified. Multiple samples of species and genotypes would aid the development of markers for the identification of *Arnebiae Radix* species as well as for assessments of the population structure of *A. euchroma* and *A. guttata*.

Here, the chloroplast genomes of 56 *Arnebiae Radix* samples and its allies (including five *Arnebia* species) were sequenced and assembled using genome skimming methods. Specifically, we aimed to (i) elucidate variation in the chloroplast genome among *Arnebia* species in China, (ii) evaluate the divergence times among *Arnebia* species, (iii) identify markers that could be used to discriminate between different *Arnebia* species, and (iv) evaluate the genetic structure of and genetic divergence between *A. euchroma* and *A. guttata*. The chloroplast genome resources presented in this study will aid the conservation and exploitation of *Arnebia* species.

## Materials and Methods

### Plant Material

Fifty-six samples covering five Arnebia species were included in this study. A total of 50 samples were collected from field in China. DNA of four samples from Russia, and two from Mongolia were acquired from the DNA bank of China in Institute of Botany, Chinese Academy of Sciences. The samples included seven genotypes of *A. decumbens*, 15 genotypes of *A. euchroma*, six genotypes of *A. fimbriata*, 17 genotypes of *A. guttata*, and 11 genotypes of *A. szechenyi*. Samples were derived from various localities in Asia and were representative of the geographical distributions of the five *Arnebia* species. Sample information is provided in Supplementary Table 1.

### Genome Sequencing and Chloroplast Genome Assembly

Total DNA was extracted using the modified CTAB method (Li et al., 2013). DNA quality was measured using a Qubit 2.0 Fluorometer (Thermo Fisher Scientific, Waltham, United States). A total of 500 ng of DNA was used for sequencing library preparation. Total DNA was sheared to 350-bp fragments using an ultrasonicator. Illumina paired-end DNA library construction and paired-end whole-genome shotgun resequencing (150 bp) on an Illumina HiSeq X-ten platform at Novogene (Tianjin, China) were performed; each sample yielded approximately 5 Gb of data.

Quality control of the raw data was conducted using Trimmomatic 0.36 (Bolger et al., 2014) with the following parameters: LEADING, 20; TRAILING, 20; SLIDING WINDOW, 4:15; MIN LEN, 36; and AVG QUAL, 20. GetOrganelle (Jin et al., 2020) with k-mer lengths of 85, 95, and 105 was used to assemble the whole chloroplast genomes. If GetOrganelle was unable to successfully assemble the whole chloroplast genome, we followed the methods of Dong et al. (2022a) to assemble the chloroplast genome sequences. Genomes were annotated using Plann (Huang and Cronk, 2015), and the published genome of *A. guttata* (GenBank Accession number: MT975391) was used as the reference sequence. Annotated whole chloroplast genome sequences were submitted to GenBank. The *Arnebia* chloroplast genomes maps were depicted using Chloroplot (Zheng et al., 2020).

### Development of Molecular Markers

All the chloroplast genome sequences were aligned using MAFFT 7 (Katoh and Standley, 2013) and adjusted manually using Se-Al 2.0 (Rambaut, 1996); for example, alignment errors associated with polymeric repeat structures and small inversions were corrected to avoid overestimation of sequence divergence. mVISTA and nucleotide diversity (π) were used to analyze interspecific variation in *Arnebia* chloroplast genomes (Frazer et al., 2004). Mutational hotspots were identified by sliding window methods; the window size was set to 800 bp, and the step size was set to 100 bp.

Nucleotide diversity, variable, and parsimony-informative sites were used to assess marker variability for hypervariable markers (mutational hotspot regions). The three universal chloroplast DNA markers, *rbcL*, *matK*, and *trnH-psbA*, were used in this analysis to compare variation between hypervariable markers and universal chloroplast DNA markers. The number of variable and parsimony-informative sites and nucleotide diversity (π) were calculated using MEGA 7.0 (Kumar et al., 2016) and DnaSP v6 (Rozas et al., 2017).

### Phylogenetic Analyses

We used all the coding genes to reconstruct the phylogenetic relationships among *Arnebia* and other Lithospermeae species at the species level. The dataset included 12 *Arnebia* samples and samples from eight other Boraginaceae species. Coding genes were extracted using Geneious Prime v2020.0.5 based on annotation of the chloroplast genomes. Nucleotide and amnio acid sequences were used for phylogenetic analyses. The whole chloroplast genome dataset of all *Arnebia* samples was used to infer the phylogenetic relationships among all genotypes.

Maximum likelihood (ML) and Bayesian inference (BI) were used to infer phylogenetic relationships. ML analyses were run using RAxML-NG (Kozlov et al., 2019) with 500 bootstrap replicates. ModelFinder (Kalyaanamoorthy et al., 2017) was used to select the best-fit model of nucleotide substitution with the Bayesian information criterion. The BI tree was generated in MrBayes v3.2 (Ronquist et al., 2012). The BI analysis was run with two independent chains and priors for 20 million generations, with sampling every 1,000 generations. The stationary phase was examined using Tracer 1.6 (Rambaut et al., 2014), and the first 25% of the sampled trees was discarded. The remaining trees were used to generate a majority-rule consensus tree to estimate posterior probabilities.

### Time Calibration of the Phylogeny

The chloroplast gene data were used to estimate the divergence times among *Arnebia* species. Two priors from the findings of (Chacón et al., 2019) were used for these analyses. The crown age of Lithospermeae was constrained to 42.5 Mya [95% highest posterior density (HPD): 35.3–51.5 Mya]. The crown age of *Onosma* and *Lithospermum* was 32.1 Mya. The two priors were placed under a normal distribution with a standard deviation of 1.

We used the whole chloroplast genome data of 56 genotypes of the five *Arnebia* species to infer divergence times at the intraspecific level. Three priors from the above results were used for this analysis: (i) the crown age of *Arnebia* (the root of the tree); (ii) the crown age of *A. guttata* and *A. decumbens*; and (3) the crown age of *A. euchroma* and *A. szechenyi*/*A. fimbriata*. All three priors were placed under a normal distribution with a standard deviation of 1.

The divergence time analyses were performed in BEAST 2 (Bouckaert et al., 2014). The prior tree Yule model and GTR model were selected with the uncorrelated lognormal distribution relaxed molecular clock model, and the Markov Chain Monte Carlo (MCMC) tool was run for 500,000,000 generations with sampling every 50,000 generations. Tracer 1.6 (Rambaut et al., 2014) was used to evaluate convergence and to ensure a sufficient and effective sample size for all parameters surpassing 200. A maximum credibility tree was generated with mean heights in TreeAnnotator, with the first 10% of the trees discarded as burn-in.

### Genetic Diversity and Population Differentiation of *Arnebia euchroma* and *Arnebia guttata*

Number of variable sites, nucleotide diversity (π), and number of haplotypes were calculated using MEGA 7.0 and DnaSP v6 within the five species to characterize intraspecific variation in genetic diversity. Filtered intraspecific SNPs were used to analyze population structure with an admixture model-based clustering method implemented in STRUCTURE v. 2.3.4 (Falush et al., 2007). Allele frequencies were assumed to be correlated among populations (*K* = 1 to *K* = 10). The most likely number of clusters was determined based on both LnP(D) and Δk. Principal component analysis (PCA) was also used to assess genetic structure. PCA was conducted using Plink (Purcell et al., 2007), and graphs were built using the ggbiplot package in R.

## Results

### Structure, Gene Content, and Sequences in Five *Arnebia* Species Chloroplast Genomes

**FIGURE 1:**
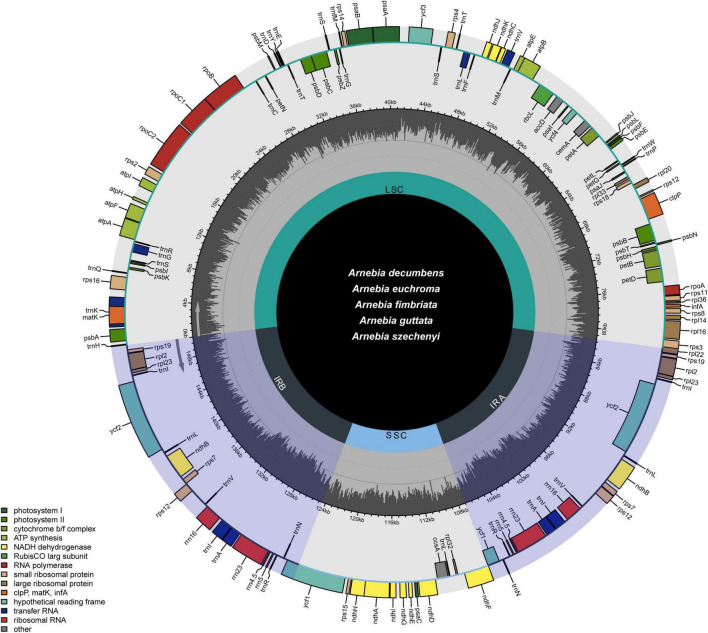
Gene map of the *Arnebia* chloroplast genome. Genes are colored according to functional categories.

The whole chloroplast genomes of the five *Arnebia* species had the quadripartite structure typical of most angiosperm species (Figure 1). The length of the 56 chloroplast genomes ranged between 149,539 and 152,040 bp (Table 1 and Supplementary Table 2). The LSC (between 80,462 and 82,946 bp) and SSC (between 17,143 and 17,336 bp) were separated by two IR regions (IRa and IRb, between 25,753 and 26,053 bp). The overall GC content was approximately 37.5–37.8%, and the GC content was slightly higher in the IR (43.0%) regions than in the LSC (35.6%) and SSC (31.3%) regions. The *Arnebia* chloroplast genome encoded 112 unique genes, including 78 protein-coding genes, 30 tRNA genes, and four rRNA genes. Eighteen genes had introns; 16 genes had a single intron, and two genes (*ycf3* and *clpP*) had two introns. There were three intron-containing genes (*ndhB*, *trnI-GAU*, and *trnA-UGC*) in the IR regions. The *matK* gene was located in *trnK*-UUU, which is the largest intron in the chloroplast genome, and the *rps12* gene was *trans*-spliced, with two copies of the 3′ end in the IR region and the 5′ end in the LSC region. The IR/SC boundaries slightly differed among the five *Arnebia* species. For example, the *trnH* gene was located in the LSC region, 12 to 42 bp from the IRa/LSC boundary.

**TABLE 1:** Chloroplast genome features of the five *Arnebia* species.

Species	Nucleotide length (bp)				GC content (%)	Number of genes			
	LSC	IR	SSC	Total		Protein	tRNA	rRNA	Total
*Arnebia decumbens*	80,462–80,826	25,943–25,945	17,188–17,203	149,539–149,919	37.8	78	30	4	112
*Arnebia euchroma*	81,086–81,708	25,918–25,968	17,256–17,336	150,224–150,900	37.6	78	30	4	112
*Arnebia fimbriata*	82,846–82,946	25,896–25,899	17,289–17,298	151,927–152,040	37.5	78	30	4	112
*Arnebia guttata*	80,737–81,265	25,967–26,053	17,143–17,213	149,840–150,410	37.7	78	30	4	112
*Arnebia szechenyi*	82,064–82,569	25,753–25,940	17,156–17,302	150,867–151,701	37.6-37.7	78	30	4	112

### Genome Variation and Molecular Marker Identification

The mVISTA results revealed high synteny among the chloroplast genomes of the five *Arnebia* species, which indicates that the chloroplast genome is highly conserved in this genus (Supplementary Figure 1). The 65 whole chloroplast genomes had an aligned length of 157,292 bp (Table 2), including 4,921 variable sites (3.13%) and 3,996 parsimony-informative sites (2.54%). The overall nucleotide diversity (π) of the chloroplast genome was 0.00634. The nucleotide diversity was lowest for the IR region (0.00177) and highest for the SSC region (0.01234). The SSC region also had the highest proportion of variable sites and parsimony-informative sites. These results indicate that the SSC region had the highest mutation rate and sequence divergence and that the IR region was more conserved compared with other regions (Table 2 and Supplementary Figure 1).

**TABLE 2:** Analyses of the variable sites in chloroplast genomes of *Arnebia*.

Regions	Aligned length (bp)	Variable sites		Information sites		Nucleotide diversity	Number of haplotypes
		Numbers	%	Numbers	%		
LSC	87,252	3,518	4.03	2,861	3.28	0.00843	49
SSC	17,664	964	5.46	791	4.48	0.01234	48
IR	26,179	214	0.82	168	0.64	0.00177	36
Whole chloroplast genome	157,202	4,921	3.13	3,996	2.54	0.00654	49

**FIGURE 2:**
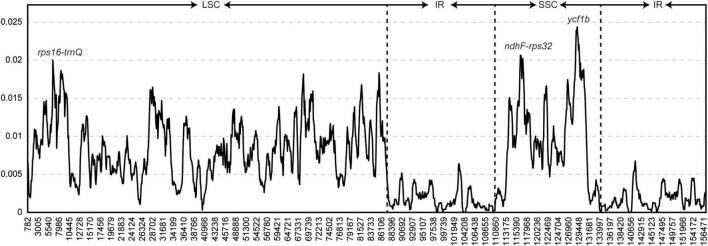
Hypervariable regions in the *Arnebia* chloroplast genome. Window length: 800 bp; step size: 100 bp. Three regions with the highest π values were marked. *x*-axis: position of the midpoint of a window; *y*-axis: nucleotide diversity of each window.

The sliding window method was used to identify mutational hotspots in the whole chloroplast genome using a window size of 800 bp (Figure 2). The π values ranged from 0 to 0.02439. Nucleotide diversity was lowest in the IR region. Three peaks were identified in the 65 whole chloroplast genomes, with π values >0.02. The three markers contained the two intergenic regions *rps16-trnQ* and *ndhF-rpl32* and the coding region *ycf1b*. *rps16-trnQ* was located in the LSC region, and *ndhF-rps32* and *ycf1b* were located in the SSC region.

We compared the number of variable sites among the three hypervariable markers and the three universal DNA barcodes (*rbcL*, *matK*, and *trnH-psbA*). Marker information is shown in Table 3. The length of the three hypervariable markers was 971 bp (*rps16-trnQ*), 1,640 bp (*ndhF-rpl32*), and 1,843 bp (*ycf1b*). *Ycf1b* had the greatest number of variable sites (153 sites), followed by *ndhF-rpl32* (125 sites) and *rps16-trnQ* (72 sites). The number of variable sites for *rbcL*, *matK*, and *trnH-psbA* was 67, 73, and 40, respectively. *Ycf1b* had the highest π value and showed more sequence divergence among the six markers, followed by *trnH-psbA* and *rps16-trnQ*. *RbcL* and *matK* were less variable according to the π values.

**TABLE 3:** Three hypervariable regions and the universal markers of chloroplast genomes of *Arnebia*.

Barcode	Number of sequences	Length (bp)	Variable sites		Information sites		Nucleotide diversity (π)
			Numbers	%	Numbers	%	
*rps16-trnQ*	56	971	72	7.42	60	6.18	0.02002
*ndhF-rpl32*	56	1,640	125	7.62	107	6.52	0.01852
*ycf1b*	56	1,843	153	8.30	130	7.05	0.02087
*trnH-psbA*	56	472	67	14.19	54	11.44	0.02019
*matK*	56	1,536	73	4.75	57	3.71	0.00936
*rbcL*	56	1,434	40	2.79	34	2.37	0.00668

### Plastid Phylogenomics of *Arnebia*

**FIGURE 3:**
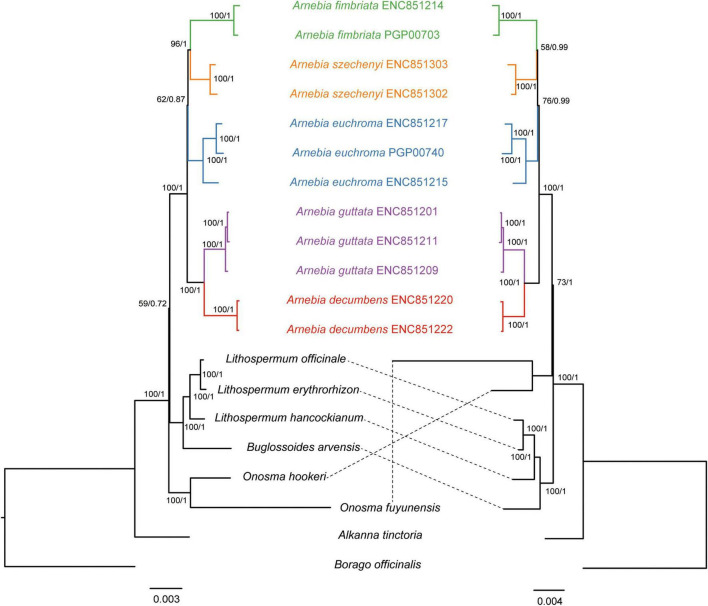
Phylogenetic trees of Lithospermeae based on all protein-coding genes. The nucleotide dataset **(left)** and amino acid dataset **(right)**. Maximum likelihood (ML) bootstrap support values/Bayesian posterior probabilities are shown at each node.

All 78 protein-coding genes of the chloroplast genome were used to infer the phylogenetic relationships among *Arnebia* species and their allies through BI and ML (Figure 3). The nucleotide data matrix contained 67,557 sites, including 4,712 variable sites and 1,675 parsimony-informative sites. The amino acid data matrix contained 22,519 sites, including 1,834 variable sites and 626 parsimony-informative sites.

The topologies of the ML and BI tree were consistent, and most nodes had strong support values. *Alkanna tinctoria* was the first group to diverge from Lithospermeae. The nucleotide dataset indicated that *Onosma* was the second group to diverge from [name]; however, the sequence of divergence inferred from the amino acid dataset differed from that inferred by the nucleotide dataset. Both datasets indicated that *Buglossoides* was sister to *Lithospermum* and formed a clade. *Arnebia*, *Buglossoides*, and *Lithospermum* formed a monophyletic group with low support values (BS = 59/PP = 0.72) based on the nucleotide dataset; the amnio acid dataset indicated that *Arnebia* was sister to *Onosma* (BS = 73/PP = 1). The inferred phylogenetic relationships among the five *Arnebia* species predicted by both datasets were similar (BS = 100/PP = 1). *A. decumbens* and *A. guttata* formed a clade with strong support values. *A. fimbriata* and *A. szechenyi* formed a clade that was sister to *A. euchroma* with moderate support values (BS = 62/PP = 0.87 or BS = 62/PP = 0.99). All the samples for each species formed a monophyletic group with high support values based on the whole chloroplast genome sequences.

### Divergence Time Estimation

**FIGURE 4:**
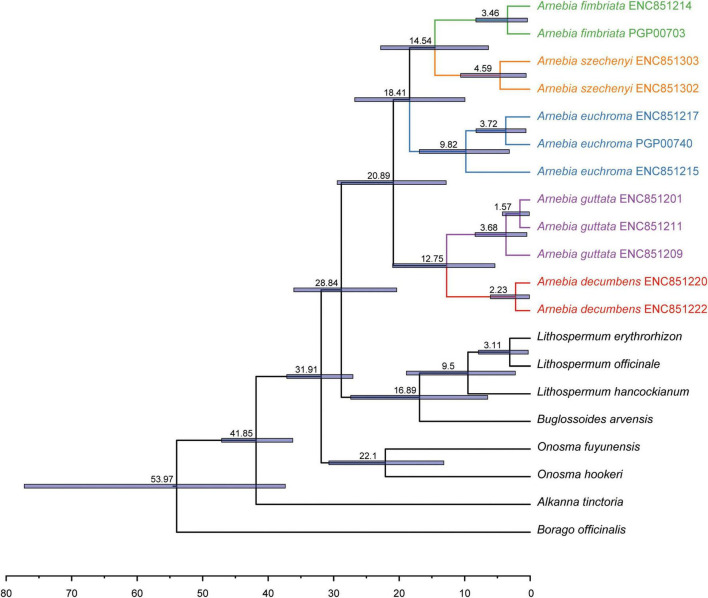
Divergence times of Lithospermeae obtained from BEAST analysis based on all coding protein-coding genes with two priors. The mean divergence time of the nodes is shown next to the nodes, and the blue bars correspond to the 95% highest posterior density (HPD).

**FIGURE 5:**
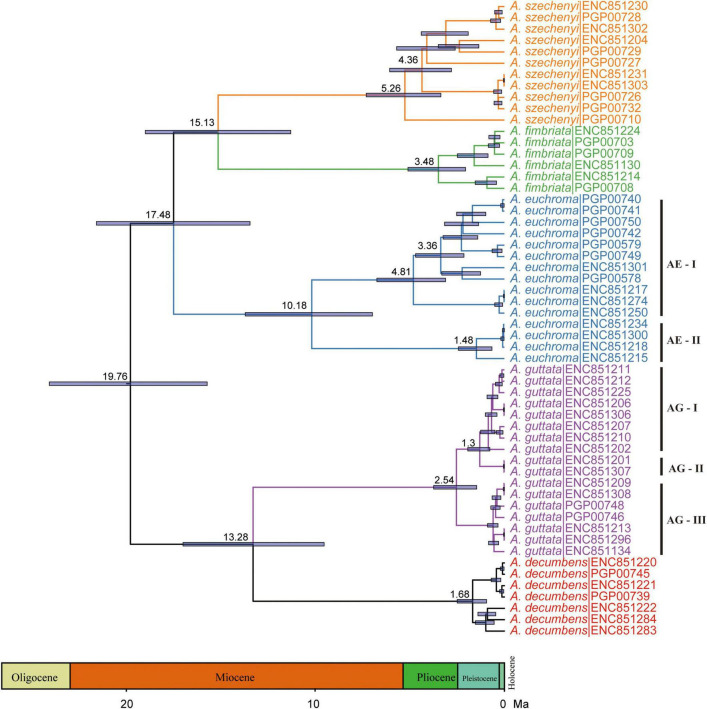
Divergence times of *Arnebia* obtained from BEAST analysis based on the 65 whole chloroplast genomes. The mean divergence time of the nodes is shown next to the nodes, and the blue bars correspond to the 95% HPD.

Divergence time estimates showed that the stem and crown nodes of *Arnebia* were 28.84 Ma (95% HPD: 20.05–35.69 Ma) in the middle Oligocene and 20.89 Ma (95% HPD: 12.82–29.46 Ma) in the early Miocene, respectively (Figure 4). The divergence time between *A. guttata* and *A. decumbens* was 12.75 Ma (95% HPD: 5.37–20.99 Ma) in the middle Miocene. The three species *A. fimbriata*, *A. szechenyi*, and *A. euchroma* diverged at 18.41 Ma, and the divergence time between *A. szechenyi* and *A. euchroma* was 14.54 Ma. The genotype-level divergence times were estimated using all samples (Figure 5). The crown ages of *A. szechenyi*, *A. euchroma*, *A. fimbriata*, *A. guttata*, and *A. decumbens* were 5.26, 3.48, 10.18, 2.54, and 1.68 Ma, respectively.

### Intraspecific Diversity and Genetic Structure of *Arnebia euchroma*

**FIGURE 6:**
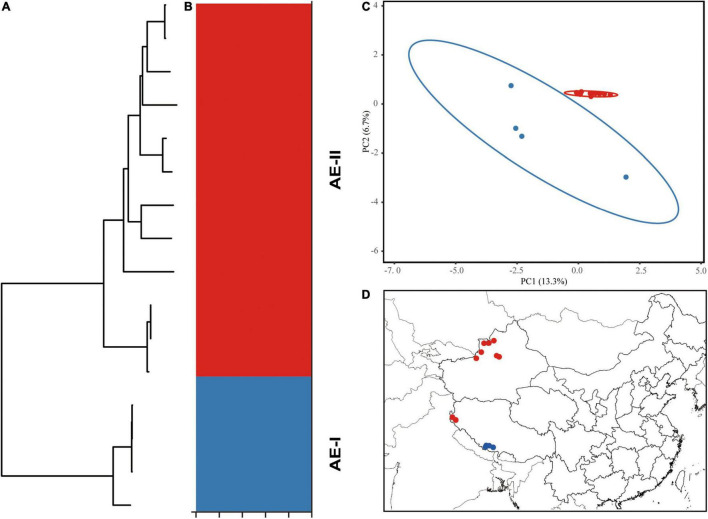
Intraspecific diversity and genetic structure of 15 *Arnebia euchroma* genotypes based on whole chloroplast genomes. **(A)** Phylogenetic tree; **(B)** population structure analysis with *K* = 2; **(C)** principal component analysis; **(D)** geographical distribution information.

The 15 aligned chloroplast genomes of *A. euchroma* were 152,175 bp in length. A total of 1,167 mutation sites were identified, including 339 singleton and 827 parsimony-informative sites (Table 4). We also identified 265 indels in the *A. euchroma* chloroplast genome. Phylogenetic analysis was performed using ML and BI and whole chloroplast genomes (Figure 6A). These samples were clearly divided into two clades (AE-I and AE-II). Population structure was analyzed using *K* values ranging from 1 to 10; the populations were clearly divided into two clades with *K* = 2 (Figure 6B). The PCA results revealed two major groups (Figure 6C). The AE-I clade included four samples located in southern Tibet (Jilong County and Zada County) (Figure 6D). The two clades exhibited significant genetic differences and diverged at 10.18 Ma in the middle Miocene (Figure 5).

**TABLE 4:** Intraspecific chloroplast genome sequence divergence of five *Arnebia* species.

Species	Number of samples	Alignment length (bp)	Number of variable sites			Nucleotide polymorphism	
			Polymorphic	Singleton	Parsimony informative	Nucleot ide diversity	Haplotypes
*Arnebia decumbens*	7	150,133	157	86	70	0.00041	7
*Arnebia euchroma*	15	152,175	1,167	339	827	0.00241	14
*Arnebia fimbriata*	6	152,386	304	135	168	0.00089	6
*Arnebia guttata*	17	150,929	313	77	236	0.00058	14
*Arnebia szechenyi*	11	152,438	709	445	259	0.00133	10

### Intraspecific Diversity and Genetic Structure of *Arnebia guttata*

**FIGURE 7:**
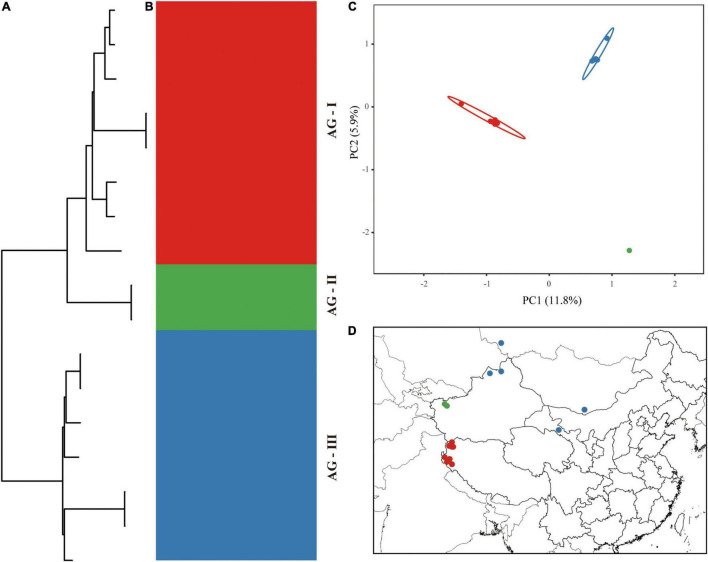
Intraspecific diversity and genetic structure of 17 *A. guttata* genotypes based on whole chloroplast genomes. **(A)** Phylogenetic tree; **(B)** population structure analysis with *K* = 3; **(C)** principal component analysis; **(D)** geographical distribution information.

The aligned 17 chloroplast genomes of *A. guttata* were 150,929 bp in length. A total of 313 mutation sites were screened, including 77 singleton and 236 parsimony-informative sites. The nucleotide diversity and number of haplotypes were 0.00058 and 14 for the 17 *A. guttata* chloroplast genome sequences, respectively. The sequence divergence among the *A. guttata* chloroplast genome sequences was lower than that among the *A. euchroma* chloroplast genome sequences (Table 4). The samples were clearly divided into three clades (AG-I, AG-II, and AG-III) according to the phylogenetic relationships (Figure 7A), STRUCTURE analysis (Figure 7B), and PCA (Figure 7C). The AG-I clade contained eight samples from Mongolia, Russia, Gansu Province, and northwestern Xinjiang, China. The AG-II clade included two samples from Tacheng, Xinjiang. The AG-III clade contained seven samples from northwestern Tibet (Figure 7D). The divergence times among the three clades ranged from 1.2 to 2.54 Ma in the Pleistocene (Figure 5).

## Discussion

### Inter- and Intraspecific Variation in the Chloroplast Genomes of *Arnebia* Species

The aim of this study was to explore patterns of chloroplast genome divergence in *Arnebia* species in China. The chloroplast genomes of the five *Arnebia* species were conserved, as little variation was observed in their size, GC content, and gene order among species. The organization of *Arnebia* genomes was similar to that of other members of the family Boraginaceae and exhibited a typical quadripartite structure. The mVISTA results and DnaSP results revealed that the LSC and SSC regions were more variable than the IR regions, and the non-coding regions were more variable than the coding regions. Mutation events were not random but clustered into hotspots in the chloroplast genome (Figure 2 and Supplementary Figure 1). Mutational hotspot regions were more variable than universal chloroplast markers (*rbcL*, *matK*, and *trnH-psbA*) or commonly used markers, such as *ndhF* and *trnL-F*. Three variable regions (*rps16-trnQ*, *ndhF-rpl32*, and *ycf1b*) were identified in the *Arnebia* chloroplast genome. *rps16-trnQ* was located in the LSC region, and this marker has long been used for the reconstruction of plant phylogenies and species identification (Smedmark et al., 2008). The *rps16-trnQ* marker contains the *rps16* intron and the intergenic spacer *rps16-trnQ* (Shaw et al., 2005; Dong et al., 2012). *rps16-trnQ* shows greater variation in the chloroplast genome. However, it contains a larger inversion in papilionoid species. The intergenic spacer *ndhF-rpl32* is located in the SSC region. This marker was identified by Shaw et al. (2007) and Dong et al. (2012), and both of these studies suggested that this marker could be used to conduct phylogenetic studies at the species or subspecies level because of its higher level of variability compared with other chloroplast markers. The *ycf1* gene is the second longest gene in the chloroplast genome, and this gene spans the IR and SSC regions. Two variable regions in this gene, *ycf1*a and *ycf1*b, have been identified (Dong et al., 2012, 2015). In the *Arnebia* chloroplast genome, *ycf1*b was the most variable marker (Figure 2 and Table 3). *ycf1* has also been shown to vary substantially among different varieties (Magdy et al., 2019; Xiao et al., 2021).

Chloroplast genome markers have long been used in plant population genetic studies, but these markers generally have few polymorphic sites. We identified many intraspecific mutations in *Arnebia* species (Table 4), and *A. euchroma* and *A. szechenyi* had a higher number of variable sites in the chloroplast genome. The polymorphic sites generate a sufficient number of haplotypes, and most of the sampled population exhibited private haplotypes. There were more variable sites in the chloroplast genomes of *Arnebia* species compared with other woody plants. Variation in the substitution rate among taxa has been examined by various studies (Smith and Donoghue, 2008; Amanda et al., 2017; Schwarz et al., 2017; Choi et al., 2021). Generation time variation is the generally accepted explanation for substitution rate variation; specifically, nucleotide substitution rates are thought to be negatively correlated with generation time (Smith and Donoghue, 2008; Amanda et al., 2017).

The genetic information contained in the chloroplast genome at the inter- and intraspecific levels can improve our understanding of plant evolution and population genetics. The rich genetic variation of *Arnebia* species can also enhance the breeding of herbal varieties.

### Phylogenetic Relationships and Divergence Times Among *Arnebia* Species

*Arnebia* species are annual or perennial plants. Johnston (1954) suggested that the genus comprises two sections: Sect. *Euarnebia*, which includes one annual species *A. tetrastigma* Forsskål, and sect. *Strobilia*, which is further subdivided into three subgroups according to its life cycle (annual/perennial) and the presence of a pubescent annulus at the base of the corolla tube. Molecular data have indicated that *Arnebia s.l.* (including *Macrotomia*) is not monophyletic (Coppi et al., 2015); however, the molecular markers used in these studies do not provide sufficient resolution for resolving species-level relationships in this group (Weigend et al., 2009; Coppi et al., 2015; Chacón et al., 2019). In this study, we used whole chloroplast genomes to infer the phylogenetic relationships among *Arnebia* species in China, and the whole chloroplast genomes provided sufficient information for resolving phylogenetic relationships at the species and subspecies level. The chloroplast genome tree did not group species by life cycle, and this finding was inconsistent with the internal transcribed spacer tree, which indicates that *Arnebia s.s.* formed a monophyletic group subdivided into two lineages corresponding to the groups of annual and perennial species (Coppi et al., 2015).

We estimated the divergence time of *Arnebia* based on the chloroplast genome at the species and subspecies levels. *Arnebia* originated from the Oligocene and diversified in the middle Miocene (Figure 4; Chacón et al., 2019). Late Oligocene warming and the progressive uplift of Tianshan and the Himalayas in the early Miocene (Favre et al., 2015) might be the main factors underlying the origin and diversification of *Arnebia.*

### Genetic Divergence of *Arnebia euchroma* and *Arnebia guttata* in China

*Arnebia euchroma* and *A. guttata* are the two source plants of *Arnebiae Radix*. Wild populations of these species have declined due to overharvesting, habitat destruction, and fragmentation (Samant et al., 1998; Qin et al., 2017). Both species are difficult to grow *via* conventional agricultural practices (Kumar et al., 2021), and this has resulted in the increased use of wild resources. Studies of genetic diversity are essential for plant seeding, agricultural practices, as well as the conservation of endangered species.

Our analyses based on the whole chloroplast genome revealed a considerably high level of genetic variation within *A. euchroma* and *A. guttata* (Table 4). Most of the samples had specific genotypes. *A. euchroma* was divided into two clades that showed significant genetic divergence (Figure 6). Divergence time estimation indicated that the two clades diverged the in late Miocene (10.18 Ma), and intraspecific divergence occurred earlier compared with other plants. We carefully examined the morphological characteristics of the populations from southern Xizang (Jilong County and Zada County) (AE-I clade), and none of them were misidentified. The AE-I clade might contain cryptic species, and more samples and field studies need to be performed in the future to explore this possibility. *A. guttata* was the source of Inner Mongolia *Arnebiae Radix*. Chinese populations comprised three subclades (Figure 7), which were consistent with the geographical distributions of this species. The divergence time of this species was estimated to be 2.54 Ma in the Pleistocene.

Several studies have shown that some herbaceous plants occurring in drylands show high levels of genetic variation (Meng and Zhang, 2011; Zhang et al., 2017; Fu et al., 2021). Ecological conditions in the drylands and the long evolutionary histories of desert plants are probably associated with the high levels of genetic variation (Meng and Zhang, 2011). The drylands and the mountains likely result in discrete patches of vegetation, which reduces gene flow and genetic similarity among populations (Maestre et al., 2012).

## Data Availability Statement

The data presented in the study are deposited in the GenBank, accession number ON529903-ON529958.

## Author Contributions

WD, LG, and LH conceived and designed the work. JS, SW, RW, KL, PQ, and LS collected the samples. JS, EL, YW, SW, RW, KL, PQ, and LS performed the experiments and analyzed the data. WD, JS, and SW wrote the manuscript. LG and LH revised the manuscript. All authors have read and agreed to the published version of the manuscript.

## Conflict of Interest

The authors declare that the research was conducted in the absence of any commercial or financial relationships that could be construed as a potential conflict of interest.

## Publisher’s Note

All claims expressed in this article are solely those of the authors and do not necessarily represent those of their affiliated organizations, or those of the publisher, the editors and the reviewers. Any product that may be evaluated in this article, or claim that may be made by its manufacturer, is not guaranteed or endorsed by the publisher.

## Supplementary Material

Supplementary Figure 1 Comparison of the five *Arnebia* species chloroplast genomes using mVISTA.
