# Supplementary figures and images for: Phylogenomics and Genetic Diversity of Arnebiae Radix and Its Allies (Arnebia, Boraginaceae) in China
Source: Front Plant Sci. 2022 Jun 9;13:920826. doi: 10.3389/fpls.2022.920826 (PMC9218939; doi:10.3389/fpls.2022.920826)

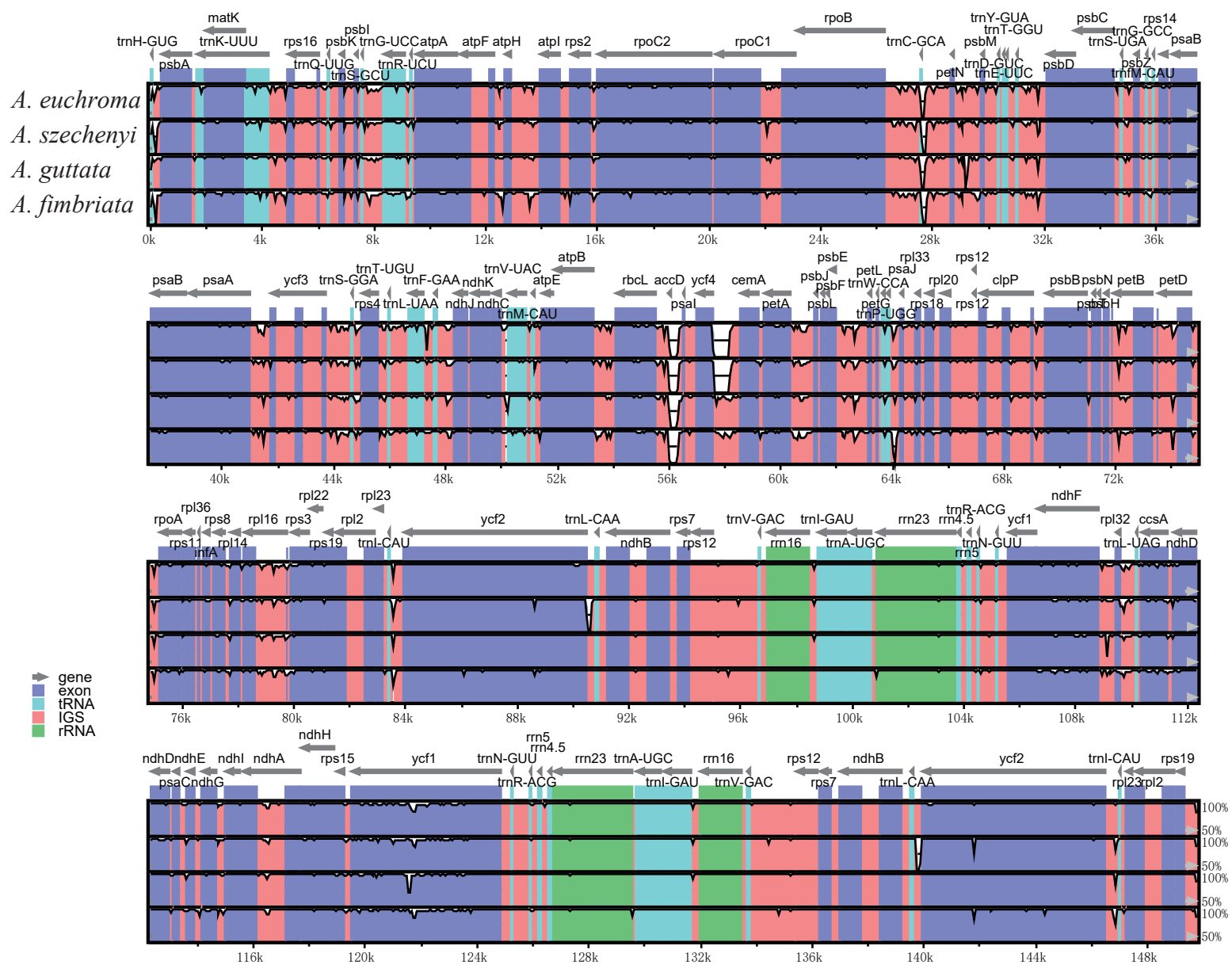

Supplement: Supplementary Figure 1 — Comparison of the five Arnebia species chloroplast genomes using mVISTA. [file Data_Sheet_1.PDF]
