# Supplementary material for: Phylogenomics and Genetic Diversity of Arnebiae Radix and Its Allies (Arnebia, Boraginaceae) in China
Source: Front Plant Sci. 2022 Jun 9;13:920826. doi: 10.3389/fpls.2022.920826 (PMC9218939; doi:10.3389/fpls.2022.920826)
Supplement: Supplementary file 2 [file Table_1.docx]

Table S1. Samples included in this study with locality, voucher, and GenBank accession numbers.

| No. | voucher | Species | Locality | GenBank accession numbers |
| --- | --- | --- | --- | --- |
| 1 | ENC851283 | *Arnebia decumbens* | Russia | ON529929 |
| 2 | ENC851284 | *Arnebia decumbens* | Russia | ON529930 |
| 3 | ENC851220 | *Arnebia decumbens* | Huocheng, Xinjiang, China, | ON529919 |
| 4 | ENC851221 | *Arnebia decumbens* | Yining, Xinjiang, China | ON529920 |
| 5 | PGP00739 | *Arnebia decumbens* | Bole, Xinjiang, China | ON529950 |
| 6 | PGP00745 | *Arnebia decumbens* | Hoboksar, Xinjiang, China | ON529954 |
| 7 | ENC851222 | *Arnebia decumbens* | Gongliu, Xinjiang, China | ON529921 |
| 8 | ENC851215 | *Arnebia euchroma* | Nielamu, Tibet, China | ON529916 |
| 9 | ENC851218 | *Arnebia euchroma* | Jilong, Tibet, China | ON529918 |
| 10 | ENC851234 | *Arnebia euchroma* | Jilong, Tibet, China | ON529926 |
| 11 | ENC851300 | *Arnebia euchroma* | Jilong, Tibet, China | ON529932 |
| 12 | ENC851217 | *Arnebia euchroma* | Zhada, Tibet, China | ON529917 |
| 13 | ENC851250 | *Arnebia euchroma* | Zhada, Tibet, China | ON529927 |
| 14 | ENC851274 | *Arnebia euchroma* | Zhada, Tibet, China | ON529928 |
| 15 | ENC851301 | *Arnebia euchroma* | Wensu, Xinjiang, China | ON529933 |
| 16 | PGP00578 | *Arnebia euchroma* | Zhaosu, Xinjiang, China | ON529939 |
| 17 | PGP00579 | *Arnebia euchroma* | Bayanbulak, Xinjiang, China | ON529940 |
| 18 | PGP00740 | *Arnebia euchroma* | Bole, Xinjiang, China | ON529951 |
| 19 | PGP00741 | *Arnebia euchroma* | Bole, Xinjiang, China | ON529952 |
| 20 | PGP00742 | *Arnebia euchroma* | Bole, Xinjiang, China | ON529953 |
| 21 | PGP00749 | *Arnebia euchroma* | Xinyuan, Xinjiang, China | ON529957 |
| 22 | PGP00750 | *Arnebia euchroma* | Yumin, Xinjiang, China | ON529958 |
| 23 | ENC851130 | *Arnebia fimbriata* | Bajanchongor-Aimag, Mongolia | ON529903 |
| 24 | ENC851214 | *Arnebia fimbriata* | Gaotai, Gansu, China | ON529915 |
| 25 | ENC851224 | *Arnebia fimbriata* | Jiuquan, Gausu, China | ON529922 |
| 26 | PGP00703 | *Arnebia fimbriata* | Dunhuang, Gansu, China | ON529941 |
| 27 | PGP00708 | *Arnebia fimbriata* | Minqin, Gansu, China | ON529942 |
| 28 | PGP00709 | *Arnebia fimbriata* | Subei, Gansu, China | ON529943 |
| 29 | ENC851209 | *Arnebia guttata* | Gorno-Altaisk, Russia | ON529910 |
| 30 | ENC851308 | *Arnebia guttata* | Gorno-Altaisk, Russia | ON529938 |
| 31 | ENC851134 | *Arnebia guttata* | Nomgon, South Gobi Province, Mongolia | ON529904 |
| 32 | ENC851202 | *Arnebia guttata* | Ritu, Tibet, China | ON529906 |
| 33 | ENC851206 | *Arnebia guttata* | Zhada, Tibet, China | ON529908 |
| 34 | ENC851207 | *Arnebia guttata* | Zhada, Tibet, China | ON529909 |
| 35 | ENC851210 | *Arnebia guttata* | Zhada, Tibet, China | ON529911 |
| 36 | ENC851211 | *Arnebia guttata* | Ritu, Tibet, China | ON529912 |
| 37 | ENC851212 | *Arnebia guttata* | Ritu, Tibet, China | ON529913 |
| 38 | ENC851225 | *Arnebia guttata* | Ritu, Tibet, China | ON529923 |
| 39 | ENC851306 | *Arnebia guttata* | Zhada, Tibet, China | ON529936 |
| 40 | ENC851201 | *Arnebia guttata* | Kashi, Xinjiang, China | ON529905 |
| 41 | ENC851307 | *Arnebia guttata* | Kashi, Xinjiang, China | ON529937 |
| 42 | ENC851213 | *Arnebia guttata* | Sunan, Gansu, China | ON529914 |
| 43 | ENC851296 | *Arnebia guttata* | Sunan, Gansu, China | ON529931 |
| 44 | PGP00746 | *Arnebia guttata* | Hoboksar, Xinjiang, China | ON529955 |
| 45 | PGP00748 | *Arnebia guttata* | Tuoli, Xinjiang, China | ON529956 |
| 46 | ENC851204 | *Arnebia szechenyi* | Alashan League, Inner Mongolia, China | ON529907 |
| 47 | ENC851230 | *Arnebia szechenyi* | Tongxin, Ningxia, China | ON529924 |
| 48 | ENC851231 | *Arnebia szechenyi* | Qingtongxia, Ningxia, China | ON529925 |
| 49 | ENC851302 | *Arnebia szechenyi* | Zhongning, Ningxia, China | ON529934 |
| 50 | ENC851303 | *Arnebia szechenyi* | Qingtongxia, Ningxia, China | ON529935 |
| 51 | PGP00710 | *Arnebia szechenyi* | Subei, Gansu, China | ON529944 |
| 52 | PGP00726 | *Arnebia szechenyi* | Litong, Ningxia, China | ON529945 |
| 53 | PGP00727 | *Arnebia szechenyi* | Pingluo, Ningxia, China | ON529946 |
| 54 | PGP00728 | *Arnebia szechenyi* | Shapotou, Ningxia, China | ON529947 |
| 55 | PGP00729 | *Arnebia szechenyi* | Xixia, Ningxia, China | ON529948 |
| 56 | PGP00732 | *Arnebia szechenyi* | Zhongning, Ningxia, China | ON529949 |
