# Supplementary material for: Phylogenomics and Genetic Diversity of Arnebiae Radix and Its Allies (Arnebia, Boraginaceae) in China
Source: Front Plant Sci. 2022 Jun 9;13:920826. doi: 10.3389/fpls.2022.920826 (PMC9218939; doi:10.3389/fpls.2022.920826)
Supplement: Supplementary file 3 [file Table_2.DOCX]

Table S2. Chloroplast genome features of the all 56 samples.

| Species | Voucher | LSC Length (bp) | IR Length (bp) | SSC Length (bp) | Complete Length (bp) | GC (%) |
| --- | --- | --- | --- | --- | --- | --- |
| *Arnebia euchroma* | ENC851215 | 81708 | 25968 | 17256 | 150900 | 37.6 |
| *Arnebia euchroma* | ENC851218 | 81474 | 25937 | 17259 | 150607 | 37.6 |
| *Arnebia euchroma* | ENC851234 | 81474 | 25937 | 17259 | 150607 | 37.6 |
| *Arnebia euchroma* | ENC851300 | 81474 | 25937 | 17259 | 150607 | 37.6 |
| *Arnebia euchroma* | PGP00742 | 81233 | 25918 | 17285 | 150354 | 37.6 |
| *Arnebia euchroma* | PGP00741 | 81206 | 25918 | 17297 | 150339 | 37.6 |
| *Arnebia euchroma* | PGP00740 | 81235 | 25918 | 17297 | 150368 | 37.6 |
| *Arnebia euchroma* | PGP00750 | 81175 | 25919 | 17298 | 150311 | 37.6 |
| *Arnebia euchroma* | PGP00579 | 81137 | 25918 | 17300 | 150273 | 37.6 |
| *Arnebia euchroma* | PGP00749 | 81086 | 25918 | 17302 | 150224 | 37.6 |
| *Arnebia euchroma* | ENC851301 | 81110 | 25924 | 17304 | 150262 | 37.6 |
| *Arnebia euchroma* | ENC851250 | 81164 | 25918 | 17305 | 150305 | 37.6 |
| *Arnebia euchroma* | PGP00578 | 81253 | 25935 | 17310 | 150433 | 37.6 |
| *Arnebia euchroma* | ENC851217 | 81183 | 25918 | 17336 | 150355 | 37.6 |
| *Arnebia euchroma* | ENC8512741 | 81183 | 25918 | 17336 | 150355 | 37.6 |
| *Arnebia decumbens* | ENC851220 | 80462 | 25944 | 17189 | 149539 | 37.8 |
| *Arnebia decumbens* | PGP00745 | 80462 | 25944 | 17189 | 149539 | 37.8 |
| *Arnebia decumbens* | ENC851221 | 80467 | 25944 | 17188 | 149543 | 37.8 |
| *Arnebia decumbens* | PGP00739 | 80469 | 25944 | 17189 | 149546 | 37.8 |
| *Arnebia decunbens* | ENC851283 | 80658 | 25945 | 17202 | 149750 | 37.8 |
| *Arnebia decunbens* | ENC851284 | 80805 | 25943 | 17200 | 149891 | 37.8 |
| *Arnebia decumbens* | ENC851222 | 80826 | 25945 | 17203 | 149919 | 37.8 |
| *Arnebia fimbriata* | ENC851130 | 82846 | 25896 | 17289 | 151927 | 37.5 |
| *Arnebia fimbriata* | ENC851224 | 82946 | 25899 | 17296 | 152040 | 37.5 |
| *Arnebia fimbriata* | PGP00703 | 82896 | 25898 | 17297 | 151989 | 37.5 |
| *Arnebia fimbriata* | ENC851214 | 82847 | 25898 | 17298 | 151941 | 37.5 |
| *Arnebia fimbriata* | PGP00708 | 82871 | 25898 | 17298 | 151965 | 37.5 |
| *Arnebia fimbriata* | PGP00709 | 82881 | 25899 | 17298 | 151977 | 37.5 |
| *Arnebia guttata* | ENC851213 | 80737 | 25980 | 17143 | 149840 | 37.7 |
| *Arnebia guttata* | ENC851296 | 80737 | 25980 | 17143 | 149840 | 37.7 |
| *Arnebia guttata* | ENC851209 | 81261 | 25980 | 17144 | 150365 | 37.7 |
| *Arnebia guttata* | ENC851308 | 81261 | 25980 | 17144 | 150365 | 37.7 |
| *Arnebia guttata* | PGP00748 | 81231 | 25980 | 17145 | 150336 | 37.7 |
| *Arnebia guttata* | ENC851134 | 81265 | 25980 | 17145 | 150370 | 37.7 |
| *Arnebia guttata* | PGP00746 | 81263 | 25981 | 17145 | 150370 | 37.7 |
| *Arnebia guttata* | ENC851201 | 81208 | 25967 | 17207 | 150349 | 37.7 |
| *Arnebia guttata* | ENC851307 | 81208 | 25967 | 17207 | 150349 | 37.7 |
| *Arnebia guttata* | ENC851202 | 81217 | 25991 | 17211 | 150410 | 37.7 |
| *Arnebia guttata* | ENC851225 | 81217 | 25973 | 17212 | 150375 | 37.7 |
| *Arnebia guttata* | ENC851211 | 81245 | 25973 | 17212 | 150403 | 37.7 |
| *Arnebia guttata* | ENC851207 | 81217 | 25973 | 17213 | 150376 | 37.7 |
| *Arnebia guttata* | ENC851212 | 81242 | 25973 | 17213 | 150401 | 37.7 |
| *Arnebia guttata* | ENC851210 | 81219 | 25974 | 17213 | 150380 | 37.7 |
| *Arnebia guttata* | ENC851206 | 80942 | 26053 | 17213 | 150261 | 37.7 |
| *Arnebia guttata* | ENC851306 | 80942 | 26053 | 17213 | 150261 | 37.7 |
| *Arnebia szechenyi* | PGP00726 | 82454 | 25934 | 17156 | 151478 | 37.7 |
| *Arnebia szechenyi* | PGP00732 | 82456 | 25904 | 17226 | 151490 | 37.7 |
| *Arnebia szechenyi* | ENC851231 | 82460 | 25934 | 17227 | 151555 | 37.7 |
| *Arnebia szechenyi* | ENC851303 | 82460 | 25934 | 17227 | 151555 | 37.7 |
| *Arnebia szechenyi* | PGP00710 | 82184 | 25928 | 17269 | 151309 | 37.7 |
| *Arnebia szechenyi* | ENC851302 | 82554 | 25934 | 17272 | 151694 | 37.6 |
| *Arnebia szechenyi* | PGP00728 | 82548 | 25940 | 17273 | 151701 | 37.6 |
| *Arnebia szechenyi* | ENC851230 | 82534 | 25934 | 17278 | 151680 | 37.6 |
| *Arnebia szechenyi* | PGP00727 | 82569 | 25926 | 17279 | 151700 | 37.6 |
| *Arnebia szechenyi* | ENC851204 | 82064 | 25753 | 17297 | 150867 | 37.7 |
| *Arnebia szechenyi* | PGP00729 | 82541 | 25894 | 17302 | 151631 | 37.6 |
